# Management of significant reactivation of old disciform scars in wet Age-Related Macular Degeneration

**DOI:** 10.1186/1471-2415-14-82

**Published:** 2014-06-25

**Authors:** Rosa M Coco, Anna Sala-Puigdollers

**Affiliations:** 1 Instituto de Oftalmobiologia Aplicada (IOBA), Campus Miguel Delibes, University of Valladolid, Pº de Belén nº 17, Valladolid 47011, Spain

**Keywords:** Age-related macular degeneration, Disciform scar, Indocyanine green angiography, Laser photocoagulation

## Abstract

**Background:**

Fibrotic disciform scars represent the end-stage of wet age-related macular degeneration (AMD) and ophthalmologists tend not to treat them. However, reactivation can occur resulting in further worsening of patients. The aim of this study is to describe the clinical outcomes of 10 patients with disciform scars from age-related macular degeneration (AMD) that have subsequently reactivated.

**Methods:**

Indocyanine green angiography (ICG) was used to identify the active areas and these “hot spots” (HS) that were subsequently treated with focal laser photocoagulation.

**Results:**

In 10 out of 11 patients with potential reactivation of an AMD scar, a treatable HS was found on the ICG at the border of the disciform scar. The identified HS was treated with focal laser photocoagulation. Post treatment these areas became inactive. However in 2 cases, reactivation occurred requiring retreatment a few months later.

**Conclusions:**

AMD patients who are noted to have disciform scars that are increasing in size and signs of activation such as lipid exudation and subretinal haemorrhage should undergo ICG imaging to look for HS. These patients could benefit from focal laser to stabilize the disease and avoid complications and further peripheral visual loss. It is suspected that these patients may have the polypoidal subtype of AMD.

## Background

Fibrotic disciform scars are typically thought to represent the end-stage of wet age-related macular degeneration (AMD) and as such ophthalmologists tend not to treat these cases [1,2]. However, reactivation can occur in the periphery of disciform scars. Mild cases may result in further worsening of patients’ central scotomas whilst severe cases can result in complications such as massive subretinal haemorrhage with sudden severe visual loss and significant impact on quality of life [3-5].

Laser photocoagulation was the first treatment available for active neovascular AMD [6]. Due to new anti-angiogenic treatments, this therapy is no longer widely used [7,8]. Laser treatment remains indicated in selected cases of AMD such as extrafoveal membranes. Similarly, a reactivation at the margin of a disciform scar is extrafoveal in location and could be amenable to focal laser treatment and it could be considered to be used for this purpose. To our knowledge this is the first report proposing the management of disciform scar reactivations using ICG and focal laser photocoagulation.

## Methods

Eleven patients with advanced wet AMD presented with a symptomatic reactivation of their longstanding fibrotic discifom scars involving the fovea. At this presentation the best-corrected distance Snellen visual acuity (VA) was under 20/200 in all patients and thus they were not considered to be treated with antiangiogenic drugs.

These patients underwent indocyanine green angiography (ICG), with 10 out of 11 having a “hot spot” (HS) identified.

All 10 patients with a HS were treated on the same day with focal laser to prevent further expansion of the lesion. Laser spots were applied using an Iridex Oculight TX 532 nm laser at 250-500 mW power. Laser pulse duration and spot size were fixed at 200 ms and 200 *μ*m respectively to mark the border of the lesion; and at 500 ms and 500 *μ*m respectively to treat the centre.

Data gathering was carried out after the Clinical Research Ethics Committee -Health Area East- of Valladolid (CEIC-VA-EAST-HCUV) approved the study protocol. This research followed the tenets of the Declaration of Helsinki.

## Results

### Patient 1

A 75-year-old female presented a reactivation of the disciform scar on her left eye (LE) with initial VA of 20/600. On examination she had a serous pigment epithelium detachment (PED) with subretinal haemorrhage and lipid exudation.

### Patient 2

A 71-year-old female presented a reactivation on her right eye (RE) with an initial VA of 20/800. She had a PED with a large area of lipid exudation. Following focal laser treatment, the area remained active 3 months later and required further focal laser treatment. Four months later, clinically the AMD appeared inactive with decrease in lipid exudation and resolution of the PED.

### Patient 3

A 65 year-old female presented a reactivation on her LE with an initial VA of 20/600. She presented with an area of retinal thickening with associated lipid exudation with no PED or subretinal haemorrhage.

### Patient 4

A 77-year-old male presented a reactivation on his LE with an initial VA of 20/600. He presented with a large serous PED with lipid exudation. The treated area showed signs of improvement at 3 months after treatment but incomplete resolution. However by 8 months, the disciform scar became inactive. A new reactivation one year later required another session of laser.

### Patient 5

A 69-year-old male presented a reactivation on his LE with an initial VA of 20/250. He presented a haemorrhagic PED.

### Patient 6

A 75-year-old female presented a reactivation on her RE with an initial VA of 20/500. She presented with a large subretinal haemorrhage with lipid exudation.

### Patient 7

**Figure 1:**
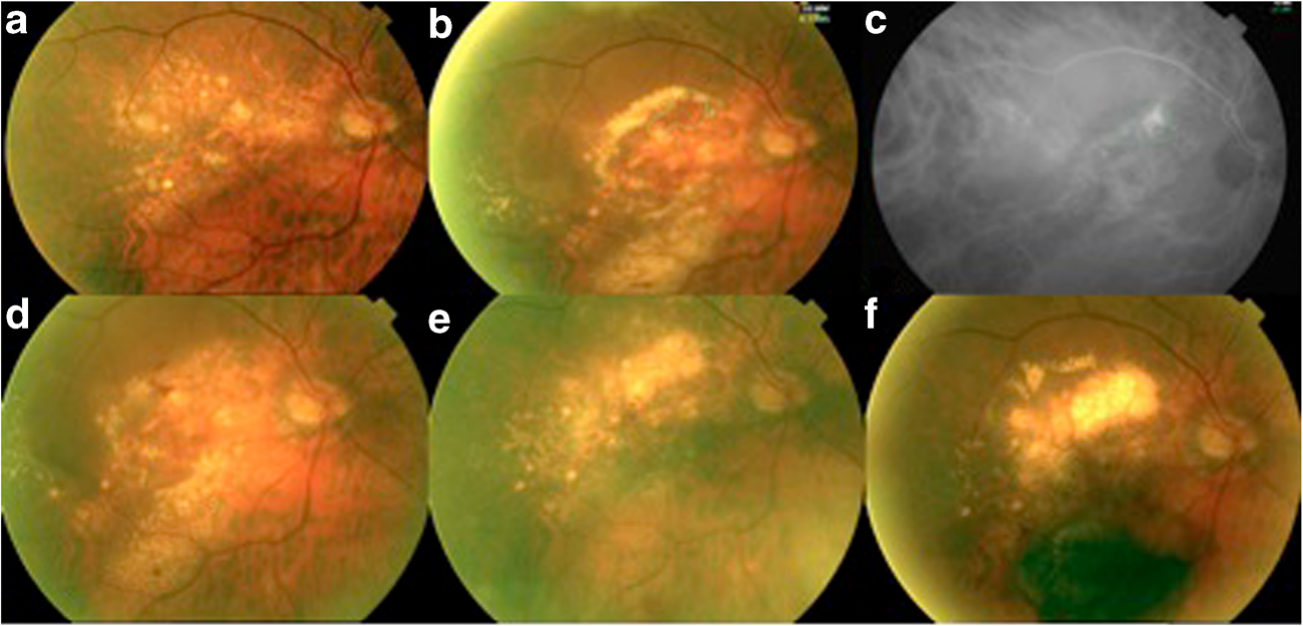
**Fundus images from Patient 7. a**. Fundus colour retinography of a disciform scar previous to the reactivation. **b**. Fundus colour retinography showing the reactivation of a disciform scar at its superotemporal margin with 2 big serous PEDs and lipid exudation. **c**. The indocyanine green angiography shows a “hot spot” **d**. Active lesion 3 months after treatment. **e**. Inactive lesion 7 month after treatment. PEDs and exudates had completely disappeared. **f**. Reactivation of a new area (haemorrage at the inferior margin) of the disciform scar one year after treatment.

A 76-year-old male presented a reactivation on his RE with an initial VA of 20/250. He presented with two serous PEDs with associated lipid exudation. The treated area remained active 3 months later, however became inactive 4 months later. At 12 months there was a new reactivation but this time involving a new margin of the disciform scar. The area previously treated remained inactive (Figure 1).

### Patient 8

An 86-year-old female presented a reactivation on her LE wit an initial VA of 20/250. She presented a large serous-haemorrhagic PED.

### Patient 9

A 79-year-old male presented a reactivation on his LE with an initial VA of 20/300. He presented with peri-papillary subretinal haemorrhages.

### Patient 10

**Figure 2:**
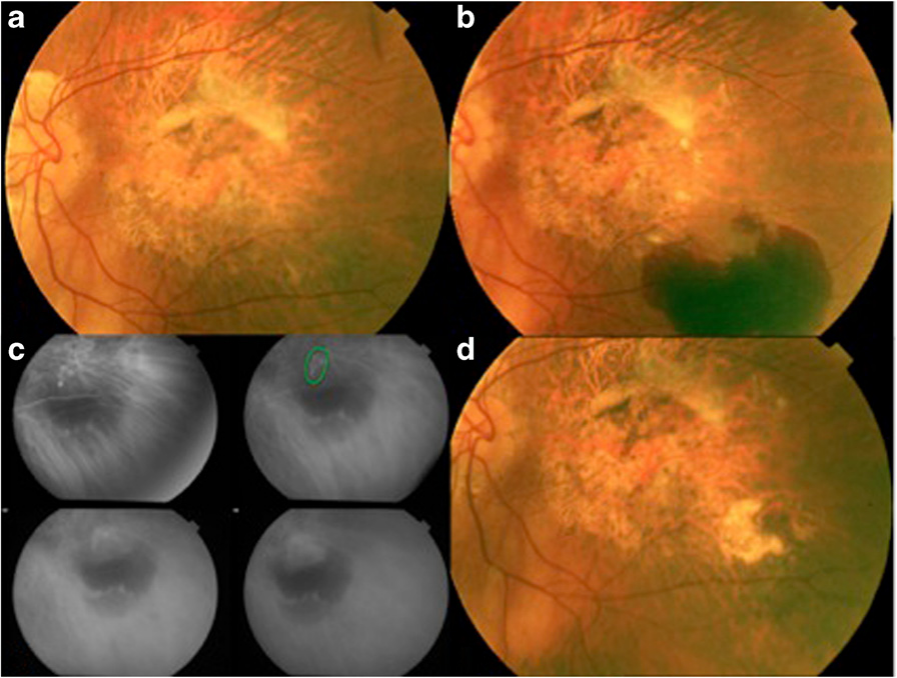
**Fundus images from patient 10. a**. Fundus colour retinography of a disciform scar previous to the reactivation. **b**. Fundus colour retinography showing the reactivation of a disciform scar at its inferotemporal margin with a big subretinal haemorrhage. **c**. The indocyanine green angiography shows a “hot spot”. **d**. Inactive lesion 9 month after treatment. The subretinal haemorrhage had completely disappeared.

A 80-year-old female presented a reactivation on her LE with an initial VA of 20/500. She presented with a large subretinal haemorrhage and PED (Figure 2).

### Long term outcome

Following this treatment, all patients were seen to have inactive AMD. Over the follow-up duration, 2 patients subsequently reactivated and needed retreatment (Table 1). VA was not seen to change following treatment.

**Table 1:** Treatment outcome of the 10 treated patients

**Patient**	**Previous treatments yes/No**	**Type and number of previous treatments (Number)**	**Period of time in which lesion became inactive after LPC (number of treatment sessions)**	**Total time of follow up remaining inactive after reaching complete inactivation**	**Reactivation**
1	No		2 months (1)	19 months	No
2	Yes	Ranibizumab IV (4)	7 months (2)	*No mo*re *follow up*	No
3	Yes	PDT (3), LPC (1)	2 months (1)	21 months	No
4	Yes	LPC (1), PDT (3), TTT (1), Ranibizumab IV(3)	8 months (1)	12 months	Yes (in the same area)
5	Yes	PDT (1), LPC (1)	3 months (1)	17 months	No
6	Yes	LPC (2), PDT (1)	3 months (1)	*No mo*re *follow up*	No
7	Yes	Ranibizumab IV (2)	7 months (1)	23 months	Yes (in a different area)
8	No		5 months (1)	27 months	No
9	Yes	TTT (1)	3 months (1)	12 months	No
10	Yes	PDT (1), Ranibizumab IV (1)	3 months (1)	9 months	No

IV: intravitreal; PDT: Photodynamic therapy; LPC: Laser photocoagulation; TTT: Transpupillary thermotherapy.

## Discussion

Age-related macular degeneration (AMD) remains one of the main causes of legal blindness among the population older than 65 years in developed countries [9]. Those AMD patients presenting a reactivation of a previously inactive disciform scar must be carefully observed. An ICG examination should be considered if there is new haemorrhage, exudate, or serous PED at the margin of the disciform scar. If a HS is seen on ICG, patients may potentially benefit from focal laser photocoagulation as this helps to stabilize the disease and possibly avoid additional complications such as massive haemorrhage [3,4], which would compromise further the quality of life of those patients. We think some of these patients, mainly the ones presenting serous or haemorrhagic PEDs, could have previously undiagnosed polypoidal lesions [10]. In such cases, periphery is important too [11]. Besides it has been described a non-negligible risk of vitreous haemorrhage after treatment with ranibizumab [12], and after PDT [13]. This risk is higher in patients under antiagregant or anticoagulant therapy [14], something common in elderly. Besides, the bigger the size of lesion (like the ones presented in this series), the worse the prognosis in polypoidal choroidal vasculopathy (PCV) [15].

It is accepted that laser controls a large number of eyes with extrafoveal PCV [16]. But we need to take into account potential complications of thermal laser photocoagulation that include formation of chorioretinal scars, secondary CNV, RPE tears, subretinal or sub-RPE hemorrhage and vitreous hemorrhage [17]. Moreover, laser has disadvantages such as higher recurrence rates [18], and enlargement of laser scars following laser treatment [19].

A significant number of patients were found to have HS in our series. On this basis, we recommend on-going following up of AMD patients with disciform scars to enable ICG-guided identification of potentially treatable reactivations. This proposal is a new variation in disease management as discifom scars tend not to be treated anymore.

Some authors proposed the use of subretinal co-application of rtPA and bevacizumab followed by repeated intravitreal anti-VEGF injections for neovascular AMD with submacular haemorrhage [3], but none of our patients had a submacular central bleeding. Instead, they had marginal reactivations of the lesion.

Concerning the possible use of anti-vascular endothelial growth factor drugs, all patients included in this series had poor vision and a longstanding fibrotic disciform scar involving the fovea, patients excluded on pivotal trials [7,20,21]. Also treatment was performed far away of the fovea and accepted treatment of PCV at the periphery includes laser [16]. Finally, poor response to anti-VEGF has been reported in this condition too.

Others proposed indocyanine green angiography-guided focal thermal laser treatment of fibrotic choroidal neovascularization, but finding the perfusing afferent arterioles to do the feeder-vessel treatment is only possible if dynamic ICG is available. Unfortunately this technology is not available at most general ophthalmological clinics [22].

## Conclusions

Performing ICG angiography should be considered in those patients with an active disciform scar in order to look for HS since the sensitivity of this test is high. This would help identify the patients who could potentially benefit from focal laser treatment, to stabilize the disease and avoid further visual loss. It is suspected these patients have the polypoidal subtype of AMD. Limitations of our study include small sample size and lack of a control arm.

## Consent

A written informed consent was obtained from all patients for publication of this case report.

## Competing interests

The authors declare that they have no competing interests.

## Authors’ contributions

AS reviewed the patient’s charts. Both authors wrote the manuscript. RMC reviewed the manuscript. Both authors read and approved the final manuscript.

## Pre-publication history

The pre-publication history for this paper can be accessed here:

http://www.biomedcentral.com/1471-2415/14/82/prepub
